# Scars That Speak: Unraveling the Oncogenic Aftermath of Pulmonary Tuberculosis—A Narrative Review

**DOI:** 10.3390/jcm15051966

**Published:** 2026-03-04

**Authors:** Cristina Cioti, Miruna Cristian Gherase, Irina Tica, Gabriela Fricatel, Elena Ciciu, Oana Cristina Arghir

**Affiliations:** 1 Internal Medicine Department, “Sf. Apostol Andrei” Emergency County Hospital, 145 Tomis Blvd., 900591 Constanta, Romania; 2 Doctoral School of Medicine, Ovidius University of Constanta, 1 University Street, 900470 Constanta, Romania; miruna.cristian@365.univ-ovidius.ro (M.C.G.); arghir_oana@yahoo.com (O.C.A.); 3 Center for Research and Development of the Morphological and Genetic Studies of Malignant Pathology—CEDMOG, Ovidius University of Constanța, 900470 Constanta, Romania; 4 Oncology Department, “Sf. Apostol Andrei” Emergency County Hospital, 145 Tomis Blvd., 900591 Constanta, Romania; 5 Nephrology Department, “Sf. Apostol Andrei” Emergency County Hospital, 145 Tomis Blvd., 900591 Constanta, Romania; 6 Clinical Pneumology Hospital of Constanta, 40 Sentinelei Str, 900002 Constanta, Romania

**Keywords:** pulmonary tuberculosis, lung cancer, post-tuberculosis lung disease, chronic inflammation, immune dysregulation, fibrotic remodeling

## Abstract

**Background:** Pulmonary tuberculosis (PTB) and lung cancer (LC) are major causes of global respiratory morbidity and mortality. Increasing evidence suggests that tuberculosis may induce persistent pulmonary alterations that extend beyond microbiological cure, potentially facilitating lung carcinogenesis. This review synthesizes current epidemiological and mechanistic evidence linking PTB to subsequent LC development. **Methods:** A structured narrative appraisal of the literature was conducted using PubMed, Web of Science, Scopus, ScienceDirect, MDPI Journals, and Google Scholar, focusing on studies published between 2020 and 2025. Eligible publications included cohort studies, meta-analyses, observational reports, and mechanistic investigations addressing the TB–LC association. Studies were thematically categorized into epidemiological evidence, pathogenic mechanisms, diagnostic challenges, and therapeutic implications. **Results:** Population-based studies consistently demonstrate a two- to threefold increased risk of LC among individuals with prior PTB, independent of smoking and other major confounders. Mechanistically, the post-tuberculous lung is characterized by chronic inflammation, oxidative stress, fibrotic remodeling, immune checkpoint activation (including PD-1/PD-L1 signaling), dysregulated microRNA expression, and metabolic reprogramming. Clinically, overlapping radiological and histopathological features may delay cancer diagnosis. A history of TB may also influence therapeutic decisions, particularly regarding immune checkpoint inhibitors due to potential infection reactivation. **Conclusions:** PTB may represent an independent risk factor for LC through sustained structural and immunological remodeling. Structured post-TB surveillance and risk-adapted screening strategies may be warranted in selected high-risk populations.

## 1. Introduction

**Figure 1:**
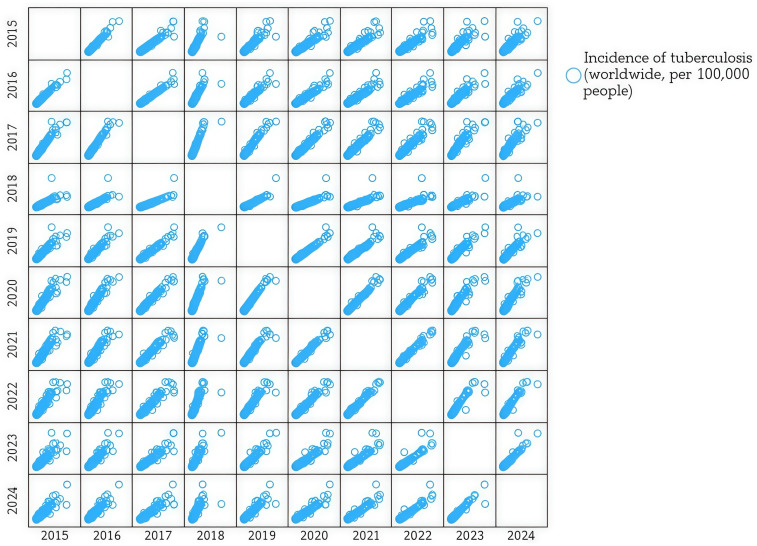
Pairwise correlation matrix of global tuberculosis incidence (2015–2024). (matrix generated in IBM SPSS version 29 from data contained in Supplementary Material S1, retrieved from Data World Bank [3].

Tuberculosis remains a major global health burden and the leading cause of death from a single infectious pathogen, with 10.6 million new cases and 1.3 million deaths reported in 2022 according to the WHO [1]. Beyond acute infection, tuberculosis is increasingly recognized as a condition capable of inducing persistent pulmonary sequelae that extend beyond microbiological cure [2]. Figure 1 shows the incidence of tuberculosis wordwide.

This matrix depicts pairwise associations of worldwide tuberculosis incidence rates (per 100,000 population) across the 2015–2024 period. The strong linear clustering observed in most panels suggests high inter-year correlation, consistent with sustained epidemiological patterns over time. The relatively tight dispersion across adjacent years indicates stability in regional TB burden distribution, whereas slightly increased variability in later years may reflect reporting disruptions or health system strain.

**Figure 2:**
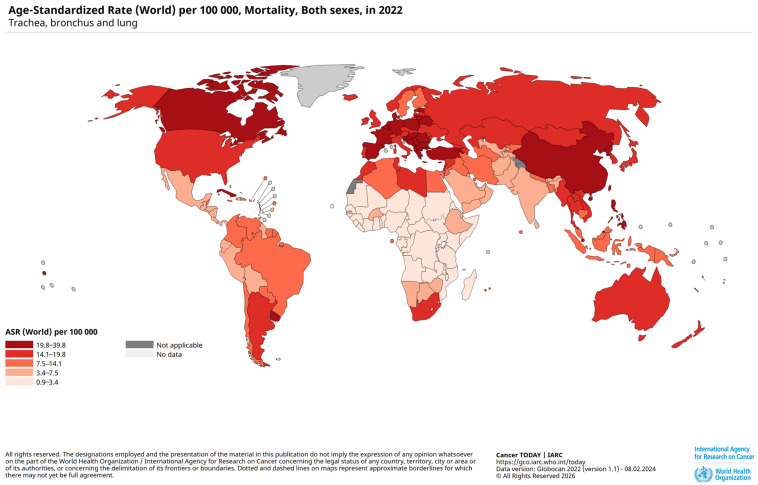
Mortality of trachea, bronchus and lung cancer in 2022. Retrieved from World Health Organization/International Agency for Research on Cancer (raw data is contained in Supplementary Material S2) [10].

Over the past two decades, large population-based studies have consistently demonstrated an increased incidence of LC among individuals with prior PTB [4,5]. This association has been reported across diverse geographic regions and healthcare systems. Meta-analyses indicate a two- to threefold higher risk of LC following PTB, independent of major confounders such as age, sex, and smoking status [6,7], supporting the hypothesis that TB represents an independent risk-associated factor for lung malignancy [8,9]. Figure 2 shows the mortality of LC wordwide.

**Figure 3:**
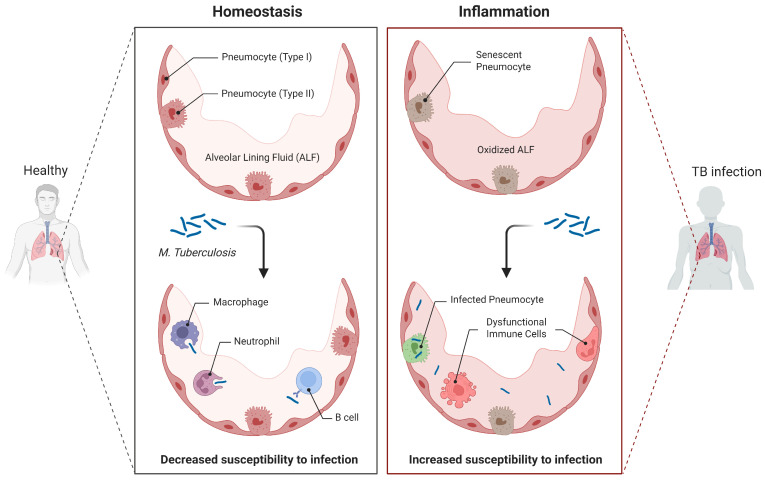
Mucosa environment increases susceptibility to M.tb. infection. Created in BioRender. (2026) https://BioRender.com/1j4tfzr (accessed on 24 February 2026) [14].

Clinically, significant overlap in symptomatology and imaging findings complicates early diagnosis. Chronic cough, hemoptysis, weight loss, and systemic symptoms are common to both conditions, particularly in endemic settings [11]. Post-tuberculous abnormalities, including fibrotic scarring, residual cavities, and nodular lesions, may radiologically mimic malignancy, increasing diagnostic uncertainty and delaying detection [12,13]. Figure 3 shows the mucosa environment of TB.

**Figure 4:**
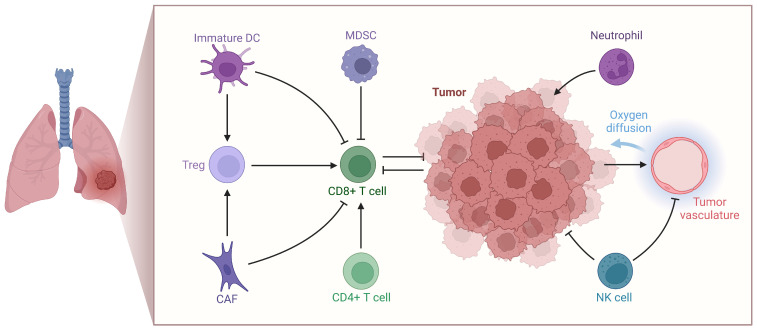
Lung tumor microenvironment. Created in BioRender. Cristina, C. (2026) https://BioRender.com/4mw8d41 (accessed on 24 February 2026) [11].

Mechanistically, chronic inflammation, oxidative stress, fibrotic remodeling, and immune dysregulation converge to create a pro-oncogenic pulmonary microenvironment [15]. Experimental and translational data suggest that these long-standing alterations promote genomic instability, epigenetic reprogramming, and impaired immune surveillance, thereby facilitating malignant transformation (Figure 4) [11].

It is important to emphasize that the association between prior pulmonary tuberculosis and subsequent lung cancer is primarily supported by observational evidence. Accordingly, the terminology throughout this manuscript has been carefully framed to reflect risk correlation rather than direct causality. While biological plausibility is strong, current epidemiological data do not allow definitive causal inference, and the observed relationship should be interpreted within the constraints inherent to non-randomized study designs. In addition, chronic obstructive pulmonary disease may act as both a mediator and a confounder in the PTB–LC association, while environmental and occupational exposures, including air pollution and silica dust, as well as socioeconomic determinants of health, may further influence risk estimates.

Despite growing evidence, the epidemiological consistency, biological plausibility, and clinical implications of this association remain incompletely integrated within a unified framework. Therefore, this narrative review systematically synthesizes current epidemiological, mechanistic, and clinical data to clarify the PTB–LC relationship and to inform future strategies for risk stratification, early detection, and structured oncological surveillance in post-tuberculous populations.

## 2. Methodology

This study was conducted in accordance with the Preferred Reporting Items for Systematic Reviews and Meta-Analyses (PRISMA) guidelines [16]. Although designed as a narrative integrative review, the literature identification and selection process followed a structured and reproducible PRISMA framework to ensure transparency in the evaluation of evidence regarding the association between PTB and LC.

A comprehensive literature search was performed for studies published between 2020 and 2025, with particular emphasis on research from the past decade to reflect contemporary advances in epidemiology and molecular oncology. Searches were conducted in PubMed, ScienceDirect and MDPI Journals.

The search strategy combined Medical Subject Headings (MeSH) terms with relevant free-text keywords, including “TB,” “lung cancer,” “post-tuberculosis fibrosis,”, “carcinogenesis,” “tuberculosis sequelae”, and “coexistence of TB and lung cancer” [17].

**Figure 5:**
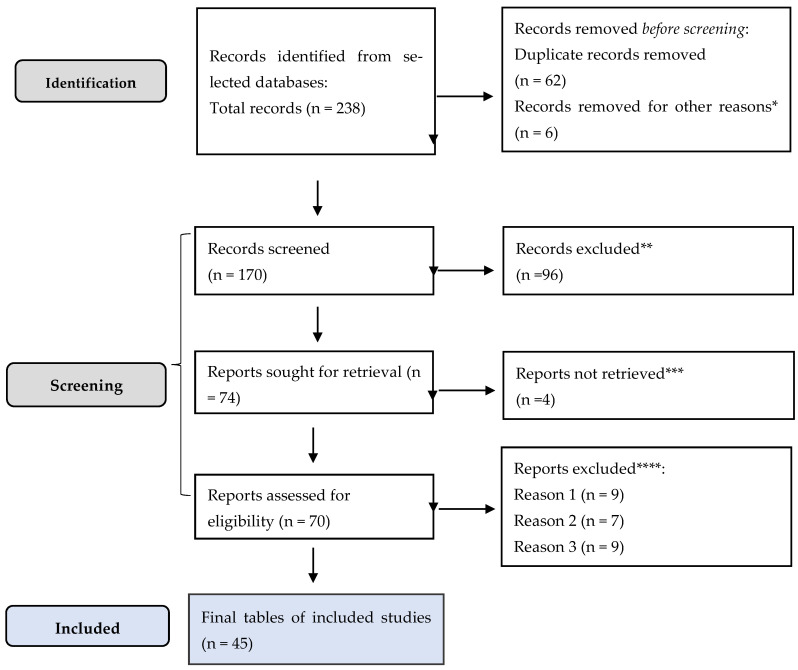
PRISMA framework. * Studies are not relevant for the present review; ** non RCT, wrong population; *** unable to find the full text of the study; **** Reason 1—study on animals/Reason 2—wrong setting/Reason 3—research question not relevant.

Eligible studies included original research articles (cohort and case–control studies). Case reports with significant illustrative relevance were also considered. Publications were limited to articles in English indexed in Web of Science database. Commentaries, opinions, studies lacking relevant methodological or clinical data, and publications exclusively addressing extrapulmonary TB without direct relevance to LC were excluded. The study selection process is presented in the PRISMA flow diagram (Figure 5). A complete list of studies included in the qualitative synthesis is provided in Supplementary Material S3.

The model presented is schematic and hypothesis-generating, integrating currently available mechanistic evidence; it does not represent a definitively validated causal pathway in humans.

Following eligibility assessment, the included studies were systematically organized into five major thematic domains: (i) epidemiological evidence supporting the TB–LC association; (ii) shared pathogenic mechanisms, including chronic inflammation, fibrotic remodeling, oxidative stress, mutagenesis, and relevant molecular alterations such as non-coding RNA dysregulation and tumor microenvironment changes; (iii) diagnostic challenges and radiological overlap; (iv) therapeutic implications; and (v) post-TB screening and surveillance strategies.

## 3. Results

### 3.1. From Epidemiology to Clinic: How TB Relates to Lung Cancer

Tuberculosis and LC remain major chronic respiratory diseases with substantial global impact, both from a public health and socioeconomic perspective. There is a coexistence of high TB prevalence and rising LC incidence, particularly in high-burden regions [18].

Clinically, TB and LC present with considerable overlap in symptomatology. Consequently, patients with residual pulmonary damage following TB may experience delayed recognition of LC, particularly in the absence of structured surveillance programs [19].

A nationwide cohort study from Republic of Korea, including more than one million individuals, reported a 2.1-fold increase in LC incidence among TB survivors compared with those without prior disease. Similar findings have been observed in Taiwan and South Africa, demonstrating consistently elevated LC incidence during long-term follow-up [20,21]. A 2021 meta-analysis further confirmed a significant association between TB and subsequent LC development, particularly adenocarcinoma and squamous cell carcinoma subtypes [7].

In the absence of standardized post-TB follow-up, many cases are diagnosed at advanced stages, adversely affecting prognosis and survival [22]. A summary of cohort studies and meta-analyses evaluating LC risk in post-tuberculous populations is presented in Table 1, with risk estimates expressed as adjusted hazard ratios (HR), odds ratios (OR), and relative risks (RR).

**Figure 6:**
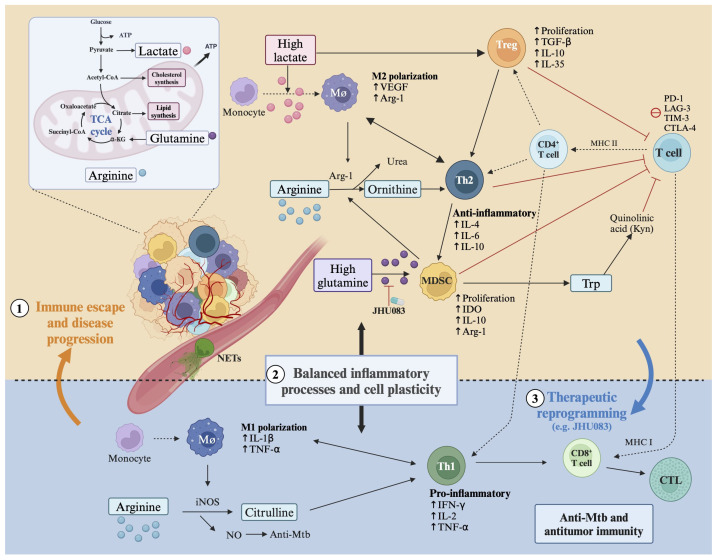
Metabolic–immune crosstalk driving tumor-permissive reprogramming in post-tuberculous lung microenvironment. Created in BioRender. Cristina, C. (2026) https://BioRender.com/quom85f (accessed on 24 February 2026) [14].

Figure 6 provides an integrative schematic representation of the principal pathogenic pathways linking TB-related lung injury to carcinogenesis, encompassing molecular alterations, immune dysregulation, and clinically relevant structural remodeling.

Elevated lactate and glutamine levels promote M2 macrophage polarization, regulatory T-cell expansion, and myeloid-derived suppressor cell activation, contributing to immune suppression and tumor progression. Arginine metabolism, IDO-mediated tryptophan degradation, and immune checkpoint signaling further reinforce T-cell dysfunction. Conversely, restoration of Th1 polarization and cytotoxic CD8^+^ T-cell activity represents a potential therapeutic reprogramming strategy.

### 3.2. Chronic Inflammation and Oxidative Stress

**Figure 7:**
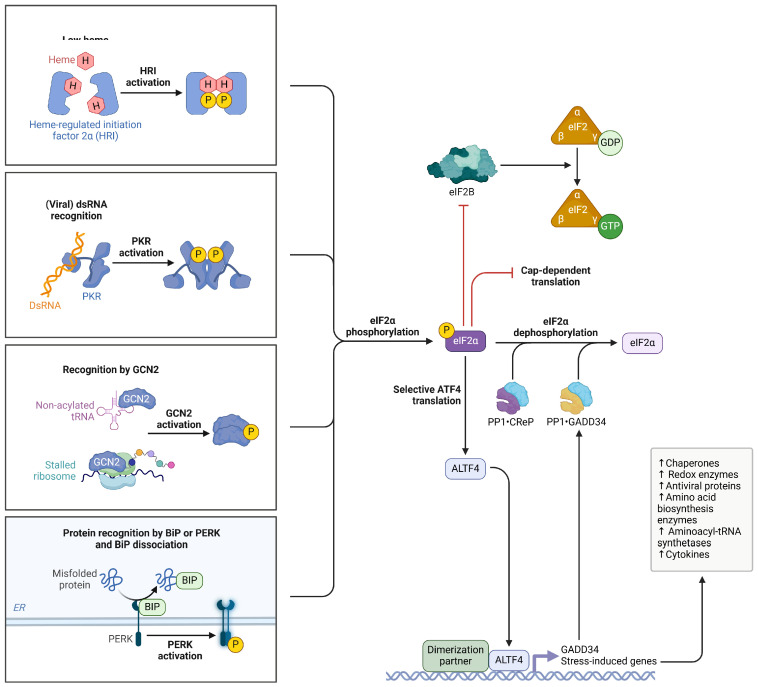
Integrated stress response pathways converging on eIF2α phosphorylation and ATF4-mediated translational reprogramming. Created in BioRender. Cristina, C. (2026) https://BioRender.com/veuhxxv (accessed on 24 February 2026) [14].

Chronic inflammation constitutes a key mechanistic link between PTB and subsequent malignant transformation. Persistent activation of innate and adaptive immune responses during MB infection results in sustained cytokine release, including interleukin-6, tumor necrosis factor-α, and interleukin-1β, maintaining a pro-inflammatory pulmonary milieu (Figure 7) [26,27].

Simultaneously, Toll-like receptor-mediated signaling promotes excessive generation of reactive oxygen and nitrogen species within alveolar macrophages and epithelial cells. Chronic oxidant imbalance induces mitochondrial dysfunction, repetitive DNA damage, and impaired repair mechanisms, thereby fostering genomic instability in previously infected tissue [28,29]. Sustained activation of inflammatory transcriptional pathways, particularly NF-κB signaling, together with persistent redox disruption, frequently coexists with fibrotic remodeling and regional hypoxia. The convergence of oxidative stress, hypoxia, and aberrant signaling drives metabolic reprogramming and immune dysfunction, creating a microenvironment permissive to malignant initiation and progression [30].

From an oncological perspective, chronic inflammation is a recognized driver of carcinogenesis, and its persistence after TB appears to amplify this risk. Population-based studies consistently report increased LC incidence in fibrotic or scarred lung regions where inflammatory activity may remain detectable long after clinical resolution [7,21,22]. Even subclinical inflammation may contribute to delayed detection and more aggressive tumor phenotypes [31].

**Figure 8:**
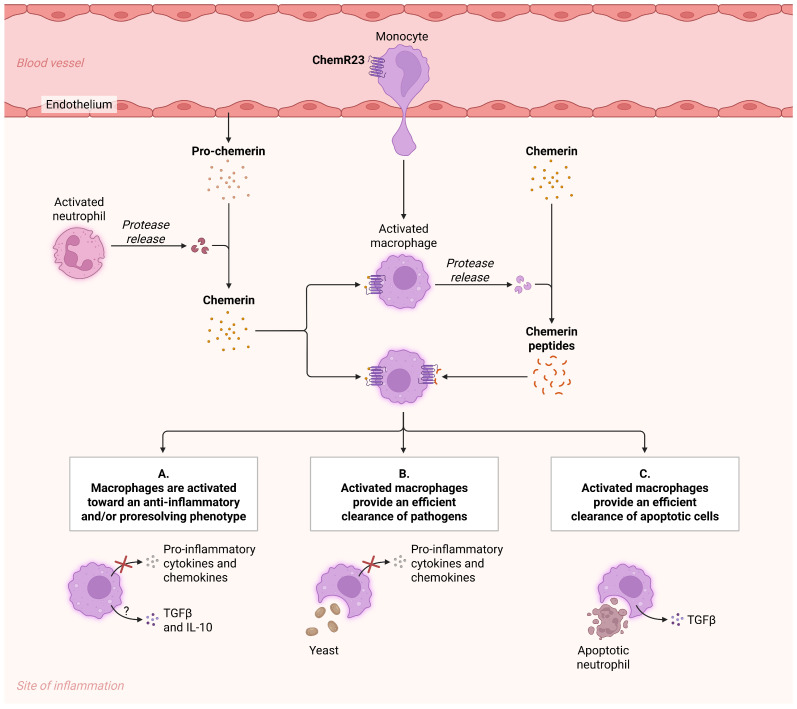
Chemerin–ChemR23 axis in chronic inflammation and tumor microenvironment remodeling. Created in BioRender. Cristina, C. (2026) https://BioRender.com/9xm7e66 (accessed on 24 February 2026) [4].

Emerging evidence suggests that the chemerin–ChemR23 (CMKLR1) signaling axis may contribute to inflammation-driven carcinogenesis through modulation of immune cell recruitment and macrophage polarization (Figure 8).

Chemerin is synthesized as an inactive precursor (prochemerin) and becomes biologically active following proteolytic cleavage by inflammatory proteases released during tissue injury. Upon binding to ChemR23 expressed on monocytes, macrophages, dendritic cells, and natural killer cells, chemerin regulates leukocyte trafficking and shapes the inflammatory microenvironment [32].

In the context of chronic pulmonary inflammation, sustained activation of this pathway promotes persistent macrophage infiltration and influence polarization toward phenotypes resembling tumor-associated macrophages. Such immune remodeling facilitates pro-inflammatory cytokine production, angiogenic signaling, and extracellular matrix reorganization. Although chemerin has been reported to exert anti-tumor effects in certain settings by enhancing natural killer cell recruitment and immune surveillance, chronic dysregulation of the chemerin–ChemR23 axis within persistently inflamed tissue supports a tumor-permissive milieu [33]. In post-tuberculous lung injury, where sustained inflammatory signaling and fibrotic remodeling coexist, prolonged activation of this pathway contributes to immune imbalance, stromal activation, and microenvironmental conditions favoring malignant transformation [34].

Thus, sustained immune activation, oxidative DNA injury, and structural remodeling provide a biologically plausible framework linking post-TB lung damage to carcinogenesis [35,36].

### 3.3. Fibrotic Remodeling and the Tumor Microenvironment

Fibrotic remodeling represents a major long-term consequence of PTB, resulting in persistent architectural distortion of the lung parenchyma. MB chronic inflammation promotes fibroblast activation, myofibroblast differentiation, and progressive extracellular matrix (ECM) deposition [37].

Post-tuberculous fibrosis creates a microenvironment characterized by hypoxia, increased ECM stiffness, and aberrant angiogenesis [38]. Sustained activation of profibrotic mediators, including transforming growth factor-β, platelet-derived growth factor, and vascular endothelial growth factor, reinforces a self-perpetuating inflammatory–fibrotic cycle.

The concept of “scar carcinoma” further supports this link, as lung cancers frequently arise within areas of chronic fibrosis and structural distortion [39]. Such regions are increasingly recognized not as passive remnants of infection but as biologically active niches that support tumor initiation and progression [40,41]. ECM stiffening and architectural remodeling may additionally impair effective immune cell trafficking, compounding local immune imbalance [42].

### 3.4. Immune Dysregulation and Local Immunosuppression

In parallel with structural remodeling, TB induces sustained immune alterations that may persist beyond infection control. Chronic antigenic stimulation maintains T-cell activation while promoting functional exhaustion, including enhanced signaling through immune checkpoint pathways such as PD-1/PD-L1 [43]. Molecular pathways including microRNA dysregulation, PD-1/PD-L1 signaling, metabolic reprogramming, and exosome-mediated communication are presented as biologically plausible and hypothesis-generating mechanisms that may contribute to tumor development in post-tuberculous lungs. However, these pathways should not be interpreted as definitively established causal mechanisms in humans.

TB is also associated with shifts in immune cell composition, including expansion of regulatory T cells and phenotypic reprogramming of macrophages and dendritic cells. These alterations impair antigen presentation and promote a chronically inflamed yet immunologically imbalanced microenvironment. In the context of fibrosis and hypoxia, such immune remodeling facilitates malignant cell persistence and immune escape [44].

Clinically, post-tuberculous lung tissue may exhibit altered tumor–immune dynamics, potentially influencing responses to immune checkpoint inhibition. Although direct mechanistic links remain under investigation, the convergence of persistent inflammation, immune exhaustion, and stromal remodeling provides a biologically coherent framework connecting TB-related lung injury to carcinogenesis.

### 3.5. Molecular, Epigenetic, and Metabolic Dysregulations in the TB–LC Context

Persistent activation of innate immune cells during TB infection, particularly macrophages and neutrophils, results in sustained oxidative stress characterized by excessive production of reactive oxygen and nitrogen species (ROS/RNS) [45]. This pro-inflammatory milieu promotes cumulative DNA damage, genomic instability, and impairment of DNA repair mechanisms, thereby facilitating activation of proto-oncogenic signaling pathways [46].

At the molecular level, non-coding RNAs have emerged as key regulators linking chronic inflammation to carcinogenesis. Altered miRNA expression in active and post-tuberculous lung tissue modulates inflammatory cascades, cell-cycle control, and epithelial proliferation, contributing to malignant transformation [47].

Concurrently, TB-associated immune remodeling promotes T-cell functional exhaustion and altered antigen-presenting cell activity, fostering an immunologically dysregulated microenvironment that facilitates tumor immune escape [48]. These molecular and immune alterations act synergistically with persistent fibrosis and chronic inflammatory signaling, collectively establishing a pro-oncogenic niche within previously injured lung parenchyma [49].

Clinical observations further indicate that LC arising in post-tuberculous lungs may exhibit distinct biological behavior, reflecting the long-term impact of infection-induced structural and immune remodeling [50].

In addition to inflammatory and immune mechanisms, TB-related metabolic reprogramming may enhance tumor susceptibility. Chronic infection shifts cellular metabolism toward aerobic glycolysis, with lactate accumulation promoting angiogenesis, immune modulation, and tumor plasticity [49]. Table 2 summarizes the pathogenic mechanisms in TB and LC.

Persistent oxidative and metabolic stress further promotes genomic instability and malignant transformation, bridging infectious pathology with neoplastic evolution [51].

### 3.6. Clinical Complexity and Diagnostic Obstacles in TB–LC Overlap

In clinical practice, the coexistence or sequential occurrence of TB and LC presents substantial diagnostic challenges due to overlapping symptoms and imaging features.

On computed tomography (CT) and positron emission tomography (PET)-CT, calcified nodules, residual cavities, and fibrotic zones may resemble neoplastic lesions, often necessitating image-guided biopsy and comprehensive histopathological, immunohistochemical, and molecular evaluation. Retrospective studies report clinically significant diagnostic delays, frequently extending several months, primarily due to radiological misinterpretation and symptom overlap [52]. Histologically, granulomatous inflammation may coexist with or obscure invasive carcinoma. Immunohistochemical markers such as TTF-1, Napsin A, and PD-L1 remain critical for accurate differentiation, although expression patterns may be altered in chronically inflamed tissue [53].

The history of TB also has therapeutic implications. Immune-based anticancer therapies, particularly immune checkpoint inhibitors, carry a risk of latent infection reactivation or exacerbation of pre-existing pulmonary damage. Therefore, prior TB should be considered an active clinical variable in oncologic decision-making, requiring careful baseline assessment and ongoing respiratory monitoring [54,55].

From a pathological standpoint, granulomatous structures composed of epithelioid cells, lymphocytes, and Langhans giant cells may coexist with LC, complicating interpretation [56]. Biopsies obtained from scarred tissue are particularly challenging, as fibrosis and caseous necrosis may mimic malignancy. Moreover, tumors developing adjacent to fibrotic scars, referred to as “scar carcinoma”, are often associated with aggressive histology [57].

Accurate diagnosis therefore requires integrative interpretation combining morphology, immunohistochemistry, imaging findings, and clinical context [58]. A structured comparison of histological and immunohistochemical features is summarized in Table 3.

Accurate differentiation between tuberculosis and LC requires integrative analysis of histological architecture, necrosis pattern, and immunohistochemical markers. In TB-endemic settings, chronic granulomatous inflammation and fibrosis can obscure malignant foci, emphasizing the importance of molecular testing and multidisciplinary correlation for accurate diagnosis and timely oncologic management [59,60].

From a therapeutic perspective, the history of TB raises important considerations regarding the selection of oncologic regimens [61,62]. Table 4 shows the evolution of microbial and inflammatory profiles across TB and LC pathologies.

### 3.7. Therapeutic Considerations and Oncological Monitoring Strategies Post-TB

**Figure 9:**
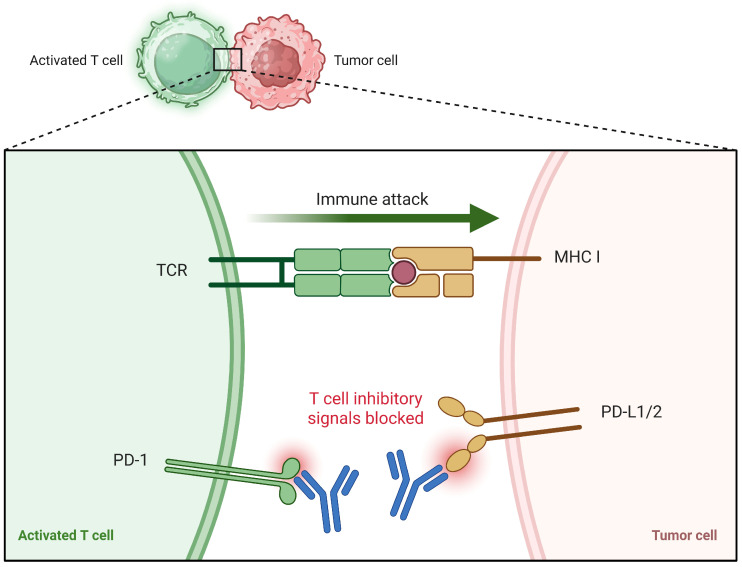
PD-1/PD-L1 immune checkpoint blockade restoring T-Cell-mediated antitumor activity. Created in BioRender. Cristina, C. (2026) https://BioRender.com/81y6m2c (accessed on 24 February 2026) [14].

Therapeutic decision-making in patients with a prior history of TB presents distinct challenges, particularly in the era of immunotherapy. Immune checkpoint inhibitors targeting the PD-1/PD-L1 axis have become central to LC management; however, their use in TB-exposed individuals raises concerns regarding reactivation of latent infection (Figure 9) [63].

Chronic TB infection promotes sustained T-cell activation and functional exhaustion, characterized by upregulation of the PD-1/PD-L1 pathway. Engagement of PD-1 on T cells with PD-L1 expressed on tumor or inflamed lung tissue transmits inhibitory signals that impair cytotoxic activity and facilitate immune escape. Immune checkpoint blockade restores T-cell-mediated antitumor responses; however, in TB-exposed individuals, this intervention may also disrupt infection–immune equilibrium, with potential implications for latent TB reactivation [64].

Case reports and observational series have documented TB reactivation during treatment with agents such as nivolumab and pembrolizumab, particularly in high-endemic regions [35].

In addition to immunotherapy-related risks, cytotoxic chemotherapy and thoracic radiotherapy may exacerbate pre-existing fibrotic sequelae of TB, potentially contributing to respiratory deterioration. Comprehensive assessment of baseline pulmonary function and careful evaluation for active or latent TB are therefore essential before initiating oncologic treatment [21,29].

Current international guidelines, including ATS/CDC/IDSA recommendations, support systematic screening for latent TB infection prior to immune checkpoint inhibition, particularly in high-incidence settings. In selected patients, prophylactic therapy with isoniazid or rifampin may be considered to mitigate reactivation risk [65].

### 3.8. Screening Strategies and Oncological Follow-Up Algorithms Post TB

Given the increased LC risk associated with prior TB, individuals with post-tuberculous lung disease may warrant risk-adapted screening strategies. Low-dose computed tomography (LDCT) should be considered in patients meeting high-risk criteria, including age > 50 years, smoking history, or extensive residual pulmonary damage. International guidelines (ATS, ERS, IDSA) support screening for selected TB survivors, particularly in high-incidence settings [66].

Emerging biomarkers, such as miR-155 and PD-L1 expression, may further refine risk stratification, although clinical validation remains ongoing [66,67]. Proposed surveillance approaches include periodic LDCT (every 6–12 months), integrated with pulmonary assessment [68].

Implementation remains challenging due to limited infrastructure, cost constraints, and the risk of overdiagnosis, especially in low-resource regions [69]. Therefore, screening should be applied within evidence-based, risk-stratified frameworks. Future studies must clarify cost-effectiveness and optimize integration of imaging and biomarker-based surveillance in post-TB populations [70].

## 4. Limitations

This work is a narrative synthesis rather than a quantitative meta-analysis and is therefore subject to potential selection and publication bias. The available evidence is largely derived from heterogeneous observational cohorts and case series, limiting causal inference. Variability in definitions of post-TB sequelae and outcome measures further restrict cross-study comparability. Also, greater interpretive weight was assigned to large population-based cohort studies and meta-analyses when discussing epidemiological risk, given their broader representativeness and statistical robustness. Smaller studies and case reports were considered primarily illustrative and hypothesis-generating. Language and database constraints may also have resulted in omission of relevant studies.

## 5. Conclusions

PTB and LC represent an increasingly recognized clinicopathological continuum with substantial epidemiological and biological relevance. TB should not be viewed solely as a resolved infectious event, but as a condition capable of inducing persistent structural and immunological alterations that may facilitate carcinogenesis. Chronic inflammation, fibrotic remodeling, oxidative stress, epigenetic changes, and microenvironmental dysregulation converge to create a tumor-permissive niche that may remain active long after microbiological cure.

Clinically, overlapping symptoms and radiological features frequently delay LC diagnosis in patients with prior TB, particularly in the absence of structured surveillance. Inflammation-associated histopathological changes may further obscure early neoplastic lesions, compounding diagnostic complexity.

Therapeutic management requires multidisciplinary coordination. While immune checkpoint inhibitors are central to LC treatment, their use in TB-exposed individuals necessitates careful evaluation due to the risk of latent infection reactivation. Conventional therapies may also exacerbate residual fibrosis, highlighting the importance of baseline pulmonary assessment.

## Figures and Tables

**Table 1:** Risk of lung cancer in patients with a personal history of pulmonary tuberculosis.

Study (Year)	Country/Region	Study Design	Population Size	Risk Estimate	Key Findings
Wu CY et al., 2011 [5]	Taiwan	Retrospective cohort	~750,000	HR 1.85 (95% CI 1.65–2.10)	Increased LC risk following PTB
Yu YH et al., 2011 [23]	Taiwan	Population cohort	~500,000	HR ≈ 2.0	Higher LC incidence after PTB
Hong S et al., 2016 [11]	South Korea	Prospective cohort	>500,000	HR ≈ 2.0	Higher LC incidence and mortality
Cabrera–Sanchez J et al., 2022 [24]	Multiple	Systematic review	>1,000,000	Pooled HR ≈ 2.1	Consistent PTB–LC association
Luczynski P et al., 2022 [6]	Multiple	Meta-analysis	>2,000,000	RR ≈ 2.2	PTB independent LC risk factor
Hwang SY et al., 2022 [25]	South Korea	Meta-analysis	>2,000,000	HR 2.18 (95% CI 1.95–2.40)	Robust pooled evidence

Legend: PTB, pulmonary tuberculosis; LC, lung cancer; CI, confidence interval. Risk estimates are adjusted for relevant confounders, including age, sex, and smoking status, where applicable.

**Table 2:** Pathogenic mechanisms in tuberculosis and lung cancer.

Pathogenic Mechanism	Effects in TB	Oncogenic Consequences in LC	References
Chronic inflammation	Persistent activation of innate immune cells with sustained release of pro-inflammatory cytokines (TNF-α, IL-6, IL-1β)	Enhanced cell proliferation, angiogenesis, and immune suppression	[19,32]
Fibrotic remodeling	Post-tuberculous parenchymal scarring and chronic tissue hypoxia	Genomic instability and facilitation of malignant transformation	[29]
Oxidative stress	Excessive ROS/RNS production leading to DNA and mitochondrial damage	Mutagenesis and activation of proto-oncogenic pathways	[37]
Molecular dysregulation (miRNAs)	Altered microRNA expression affecting post-transcriptional gene regulation	Dysregulated inflammatory and proliferative signaling	[19]
Immune dysregulation	T-cell exhaustion and impaired antigen presentation	Tumor immune evasion and disease progression	[31,32]
Metabolic reprogramming	Shift toward aerobic glycolysis with lactate accumulation	Tumor plasticity, angiogenesis, and growth advantage	[39]

Legend: ROS, reactive oxygen species; RNS, reactive nitrogen species; DNA, deoxyribonucleic acid; miRNA, microRNA; TNF-α, tumor necrosis factor-alpha; IL-6, interleukin-6; IL-1β, interleukin-1 beta.

**Table 3:** Morphology and immunohistochemistry in the differential diagnosis of tuberculosis versus bronchopulmonary carcinoma.

Feature	PTB	LC	References
Chronic inflammation	Necrotizing granulomas with Langhans giant cells	Nonspecific peritumoral lymphocytic infiltrate	[42]
Necrosis	Caseous necrosis	Focal tumor necrosis	[40,42]
TTF-1	Negative or weak, non-specific expression	Positive in adenocarcinoma	[40]
Napsin A	Absent	Positive in adenocarcinoma	[40]
PD-L1	Variable expression, increased in active PTB	Clinically relevant for immunotherapy selection, often overexpressed	[31,42]
Inflammatory infiltrate	Widespread, lymphocyte- and histiocyte-predominant	Focal, heterogeneous, may mimic inflammation	[40,42]
Cellular architecture	Granulomatous organization	Glandular or solid structures with marked cytological atypia	[40,43]

Legend: TTF-1, thyroid transcription factor-1; PD-L1, programmed death-ligand 1.

**Table 4:** Evolution of pulmonary microbial and inflammatory profiles across pulmonary tuberculosis-related lung pathology and lung cancer.

Component	Healthy Lung	Active PTB	Post-PTB	LC
Microbial diversity	High	Reduced	Decreased/altered	Severely reduced
Characteristic microbiota	Prevotella, Veillonella	Streptococcus, Pseudomonas	Actinomyces, Neisseria	Fusobacterium, Veillonella
Inflammation	Physiological	Elevated	Persistent	Exaggerated, tumor-associated
Epithelial barrier	Intact	Impaired	Remodeled	Disrupted

## Data Availability

No new data created.

## Supplementary Materials

The following supporting information can be downloaded at https://www.mdpi.com/article/10.3390/jcm15051966/s1, Supplementary Material S1 (Data, Metadata countries, Metadata Indicators); Supplementary Material S2 (Mortality data for LC), Supplementary Material S3 (PRISMA reference list).

## Abbreviations

TB	Tuberculosis
PTB	Pulmonary tuberculosis
LC	Lung cancer
PTLD	Post-TB lung disease
LDCT	Low-dose computed tomography
ROS	Reactive oxygen species
RNS	Reactive nitrogen species
ECM	Extracellular matrix
Tregs	Regulatory T cells
PD-1	Programmed cell death protein 1
PD-L1	Programmed death-ligand 1
TLR	Toll-like receptor
miRNA	MicroRNA
IHC	Immunohistochemistry
TTF-1	Thyroid transcription factor 1
ATS	American Thoracic Society
ERS	European Respiratory Society
IDSA	Infectious Diseases Society of America
LTBI	Latent TB infection
